# Oral appliance treatment outcome can be predicted by continuous positive airway pressure in moderate to severe obstructive sleep apnea

**DOI:** 10.1007/s11325-017-1578-2

**Published:** 2017-10-24

**Authors:** Anders Storesund, Anders Johansson, Bjørn Bjorvatn, Sverre Lehmann

**Affiliations:** 1 Department of Thoracic Medicine, Center for Sleep Medicine, Haukeland University Hospital, Bergen, Norway; 2 Department of Clinical Dentistry–Prosthodontics, Faculty of Medicine and Dentistry, University of Bergen, Bergen, Norway; 3 Department of Global Public Health and Primary Care, University of Bergen, Bergen, Norway; 4 Department of Clinical Science, University of Bergen, Bergen, Norway

**Keywords:** Obstructive sleep apnea, Oral appliances, Continuous positive airway pressure, Apnea-hypopnea index, Treatment outcome, Prediction

## Abstract

**Background:**

Studies show that the therapeutic CPAP pressure is associated with oral appliance (OA) treatment outcome in obstructive sleep apnea (OSA) patients. However, these studies included either CPAP adherent patients using fixed pressure devices, or partly CPAP non-adherent patients using fixed pressure or auto-adjusting (auto-CPAP) devices. In many countries, auto-CPAP is predominately used, and only those non-adherent to therapy need a change to OA. Therefore, studies examining the relationship between CPAP pressures and OA treatment outcome should focus on patients non-adherent to auto-CPAP.

**Purpose:**

The purpose of this paper is to assess if CPAP pressures predicted OA treatment outcome in patients non-adherent to auto-CPAP therapy.

**Methods:**

The OA treatment responders and non-responders were defined by two success criteria ((1) AHI < 5; (2) 5 ≤ AHI < 10 and > 50% AHI reduction). Logistic regression analyses were performed for CPAP pressures and baseline variables. ROC curve analyses were used to identify CPAP pressure cutoff values, alone and combined with other explanatory variables, predicting the OA treatment outcome.

**Results:**

Eighty-seven patients with moderate or severe OSA were included. Maximum CPAP pressures (CPAPmax) were higher in non-responders by both criteria and were, together with baseline AHI, associated with the OA treatment outcome in multivariate regression analyses. ROC curves identified an optimal CPAPmax cutoff of 12 cm H_2_O, corresponding to a positive predictive value (PPV) of 0.85 in predicting non-response using criterion 1. A prediction model combining CPAPmax > 12 and baseline AHI ≥ 30 had a PPV of 1.0 for non-response by both criteria.

**Conclusions:**

Maximum CPAP pressure was a moderate predictor of OA treatment outcome, but combined with baseline AHI, the ability to identify OA non-responders was high.

## Introduction

Obstructive sleep apnea (OSA) is a common condition in which the upper airways intermittently collapse during sleep, causing partial or total cessation of airflow. This results in fragmentation of sleep and oxygen desaturations and is associated with neurocognitive impairment and an increased risk of cardiovascular disease and all-cause mortality [1, 2]. The prevalence of OSA in a large Norwegian adult population has been estimated to 8% for AHI ≥ 15 [3]. Such findings imply that OSA could have an effect on public health. Continuous positive airway pressure (CPAP) has been considered as the gold standard treatment, as it alleviates obstruction, and is shown to reduce the negative consequences of OSA [4]. However, the patient adherence is often poor, reducing the efficacy of CPAP [5]. Oral appliance (OA) is a device that maintains the upper airways patency preventing collapse and is a treatment alternative to CPAP in mild to moderate OSA, or when CPAP treatment fails [6]. OA is in general inferior in terms of reducing OSA parameters based on polysomnography [7], and around one third of these patients will have minimal improvement [8]. Nevertheless, OA treatment might be as effective as CPAP in mild to moderate sleep apnea, if titrated sufficiently [9]. Furthermore, OA adherence may be higher than for CPAP suggesting similar health outcomes on a group level for the two treatment modalities [10]. Thus, predictors of the OA treatment response are desired, preferably using unsophisticated measurements that are available as part of the daily clinical practice.

Various clinical and polysomnographic characteristics, like younger age, female gender, less obesity, position dependent OSA, and lower AHI, have been reported to correlate with OA treatment success [11–13]. The results, however, may be difficult to implement in a clinical setting, as they are not always consistent [12, 13], and because they rarely propose cutoff levels that precisely discriminate responders from non-responders, but merely lend support to the decision making when selecting therapy.

The pressure data from previous CPAP therapy is easily accessible. A therapeutic CPAP pressure > 10.5 cm H_2_O has been associated with OA treatment failure in a Japanese population [14], whereas a CPAP pressure ≥ 13 cm H_2_O predicted treatment failure in Australian patients [15]. In a Canadian study, a therapeutic CPAP pressure ≤ 9 cm H_2_O was associated with OA treatment success [16]. The two former studies selected data from fixed pressure CPAP users with acceptable adherence, whereas the latter study included both fixed pressure and auto-CPAP users, with 50% of the subjects non-tolerant to previous CPAP. In Norway, as in many other countries, the vast majority of subjects with moderate and severe OSA start with auto-adjusting CPAP machines, and only the non-adherent cases are in need of modality change to a second line treatment, such as OA.

Thus, we sought to assess the relationship between the delivered auto-CPAP pressures and the OA treatment response in a Norwegian sleep clinic population, among patients with moderate and severe OSA, all non-adherent to CPAP.

## Material and methods

### Patients

In this retrospective study, the patients were consecutively recruited from the Center for Sleep Medicine at the Haukeland University Hospital, Bergen, Norway, as described in a previous paper [17]. In brief, inclusion criteria were patients diagnosed with moderate or severe obstructive sleep apnea, non-adherent to CPAP therapy and subsequently treated with oral appliance. Non-adherence was defined as less than 4 h usage per night during a period of at least 3 months [18]. Exclusion criteria were central sleep apnea and contraindications to oral appliance therapy (e.g., poor dental status).

### Study protocol

The study protocol was approved by the Regional Committees for Medical and Health Research Ethics (number 2015/2225). All subjects had a sleep study performed at baseline before starting CPAP and attended the follow-up appointment including a new sleep study using the OA. Data on body mass index (BMI) and AHI were retrieved from the patients’ medical records and sleep polygraphy, retrospectively.

### Respiratory polygraphic evaluation

The baseline diagnosis of OSA and follow-up investigations were performed by respiratory medicine specialists or ENT specialists at the Departments of Thoracic Medicine and Otolaryngology at Haukeland University Hospital, Bergen, Norway, supported by a medical examination that included polygraphy (Embletta™, Resmed Ltd., Australia or NOX-T3®, Nox Medical, Iceland). Scoring rules were in accordance with the 2007 American Academy of Sleep Medicine manual [19].

### CPAP

All patients were treated with Resmed CPAP devices in the Autoset mode. The initial pressure settings were 4–20 cm H_2_O. CPAP compliance, expressed as the mean number of hours and minutes machine use every 24 h, and delivered CPAP pressures were recorded using CPAP software (ResScan, ResMed Corp. San Diego, CA, USA). The mean value (cm H_2_O) of the maximum CPAP pressures (CPAPmax) from each treatment session was used in the subsequent analyses. The software from ResMed auto-CPAP machines has exhibited a good diagnostic accuracy to identify residual sleep apnea, delivered pressure and mask leakage curves [20].

### Oral appliance

Maxillary and mandibular impressions and an occlusal protrusive wax or silicone index using George Gauge bite fork™ were made for all subjects, with a baseline fitting index at 50–80% of maximum protrusive capacity. The majority of patients received a titrable twin-block oral appliance while a few subjects were treated with monoblock appliances [17]. The first evaluation of subjective treatment effect was performed 4–8 weeks after starting OA. If not satisfactory, titrations were performed until positive subjective effect or until maximum adjustments had been done, after which an overnight polygraphy was performed.

### Defining treatment outcome

Two different criteria were applied when defining successful outcome with oral appliance: criterion 1 AHI < 5 and criterion 2 5 ≤ AHI < 10 and > 50% reduction from baseline.

### Statistical analysis

Comparisons between the OA outcome groups were made using student’s *t* test or Mann-Whitney *U* test for continuous variables, and chi-square test or Fisher’s exact test for categorical variables. Normality was assessed using the Shapiro-Wilk test. Anthropometrical and polygraphic data associated with the OA treatment outcomes from univariate analyses were added to the delivered CPAP pressures as explanatory variables in multivariate logistic regression models. Receiver operating characteristic (ROC) curve analyses were performed to identify CPAP pressure values with the highest sensitivity and specificity related to the OA treatment outcome. Other variables associated with OA failure from the multivariate regression models were added to CPAP pressures in the ROC curves, to assess their combined predictive utility. *P* values less than 0.05 were considered statistically significant. IBM SPSS (Statistics for Windows, version 23.0, IBM Corp., Armonk, NY, USA) was used for statistical analyses.

## Results

During the years 2007–2013, a total of 116 patients (68% males), predominantly Caucasians, were identified with a baseline diagnosis of moderate (75%) or severe OSA, who had received OA treatment due to CPAP non-adherence. Among these, 29 patients (25%) were lost to follow up for various reasons: 17 subjects had no available CPAP data, 7 did not show up for follow-up appointment, 3 died, and 2 had their follow-up elsewhere, leaving 87 patients to be included in the final analyses. Among the 87 patients, 30% had severe OSA. The patients lost to follow up did not differ from those included in this study, whose baseline and CPAP pressure characteristics are described in Table 1. The reasons for discontinuing CPAP are shown in Table 2.

**Table 1:** Anthropometrical, polygraphic, and CPAP data for all patients and according to oral appliance treatment outcome

	All patients	Criterion 1 AHI < 5		Criterion 2 5 ≤ AHI < 10 and > 50% AHI reduction	
		Responders	Non-responders	Responders	Non-responders
Number	87	31	56	56	31
Sex (male/female)	59/28	21/10	38/18	37/19	22/9
Age (years)	56.7 ± 11.7	55.5 ± 8.7	57.4 ± 13.1	55.3 ± 11.1	59.3 ± 12.5
BMI (kg/m^2^)	28.6 ± 4.2	28.2 ± 3.8	28.8 ± 4.4	28.7 ± 3.9	28.5 ± 4.7
OSA, severity (moderate/severe)	61/26	25/6	36/20	44/12*	17/14
AHI, baseline	23.8 (19.6–32.2)	20.8 (18.0–26.0)*	24.8 (20.3–39.1)	21.2 (18.2–26.9)**	28.3 (22.4–45.5)
AHI, oral appliance	6.9 (4.0–13.7)	3.1 (1.4–4.1)**	10.9 (7.1–20.0)	4.9 (3.0–6.8)***	18.2 (13.5–30.6)
AHI, CPAP	3.8 (1.9–7.7)	3.1 (0.8–9.3)	4.4 (2.2–7.7)	3.4 (2.0–5.6)	5.5 (1.6–10.7)
Maximum CPAP pressure, cm H_2_O	11.2 ± 2.8	10.1 ± 2.9*	11.7 ± 2.6	10.5 ± 2.6**	12.3 ± 2.9

Data are presented as numbers, mean ± standard deviation or median (25–75%)

*BMI*, body mass index; *OSA*, obstructive sleep apnea; *AHI*, apnea-hypopnea index; *CPAP*, continuous positive airway pressure

*p < 0.05 responders versus non-responders; **p < 0.01 responders versus non-responders; ***p < 0.001 responders versus non-responders

**Table 2:** Reasons for discontinuation of continuous positive airway pressure (CPAP)

	No. (%) (*n* = 87)
Noise or discomfort of machine	26 (30)
Claustrophobia	20 (23)
Insomnia	17 (19)
Side effects (e.g., headache, sinusitis)	12 (14)
Practical issues (e.g., travelling, power supply)	6 (7)
Tears it off during sleep	4 (5)
Unknown	2 (2)

The treatment success rates were 36% by criterion 1 and 64% by criterion 2. The CPAP median usage time (hours/night) (25–75 percentile) for all subjects was 1.2 (0.5–2.2) and did not differ between the outcome groups by any criterion (results not shown).

The OA non-responders had significantly higher CPAPmax than responders (Table 1). Using univariate logistic regression analyses, the CPAPmax was a predictor of the OA outcome by both success criteria, with an odds ratio [95% CI] = 1.25 [1.04–1.49] by criterion 1 and 1.30 [1.07–1.58] by criterion 2. When adjusted for other baseline variables in multivariate regression analyses, CPAPmax retained its ability to discriminate responders from non-responders (Table 3).

**Table 3:** Oral appliance treatment outcome predicted by maximum CPAP pressure (cm H_2_O), anthropometrical data, and polygraphic variables. Multivariate logistic regression analyses

	Criterion 1 AHI < 5		Criterion 2 5 ≤ AHI < 10 and > 50% AHI reduction	
	*p*	Odds ratio (95% CI)	*p*	Odds ratio (95% CI)
Sex	0.74	0.83 (0.28–2.48)	0.86	1.11 (0.34–3.64)
Age	0.35	1.02 (0.98–1.07)	0.05	1.05 (1.00–1.10)
BMI	0.43	1.05 (0.93–1.18)	0.99	1.00 (0.88–1.13)
AHI, baseline	0.02	1.07 (1.01–1.13)	0.001	1.09 (1.04–1.14)
CPAP maximum pressure	0.02	1.27 (1.05–1.55)	0.006	1.38 (1.10–1.74)

*BMI*, body mass index; *AHI*, apnea-hypopnea index; *CPAP*, continuous positive airway pressure

**Fig. 1:**
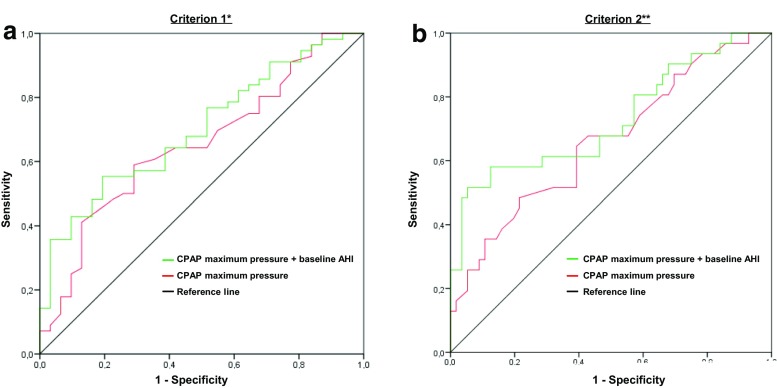
Receiver operating characteristics (ROC) curves for CPAP maximum pressure alone and in combination with baseline AHI, using oral appliance (OA) treatment success criterion 1* and criterion 2** as outcome variables. **a** Criterion 1 (AHI < 5): The area under the curve (AUC) for CPAP maximum pressure was 0.65 (0.53–0.77, *p* = 0.02) versus 0.70 (0.59–0.81, *p* = 0.002) for a prediction model combining CPAP maximum pressure with baseline AHI. **b** Criterion 2 (5 ≤ AHI < 10 and > 50% reduction from baseline): The area under the curve (AUC) for CPAP maximum pressure was 0.66 (0.54–0.78, *p* = 0.013) versus 0.73 (0.61–0.85, *p* < 0.001) for a prediction model combining CPAP maximum pressure with baseline AHI

Calculating the ROC curves for CPAPmax by criterion 1 and 2, the areas under the curve indicated moderate ability to discriminate between responders and non-responders (Fig. 1). Negative and positive predictive values, as well as likelihood ratios were calculated for different CPAP cutoff pressures (Table 4). Using criterion 1, a 12 cm H_2_O cutoff had a positive predictive value of 0.85 in predicting non-responders, involving 39% of the study population. No such cutoff pressure was identified by criterion 2, even though a CPAPmax > 14 cm H_2_O correctly classified a larger proportion of the study population.

**Table 4:** The ability of different maximum CPAP pressure levels to discriminate oral appliance treatment responders from non-responders

AUC (95% CI)	Criterion 1 AHI < 5					Criterion 2 5 ≤ AHI < 10 and > 50% AHI reduction				
	0.65 (0.53–0.77)*					0.66 (0.54–0.78)*				
Cutoff, cm H_2_O	> 10	> 11	> 12	> 13	> 14	> 10	> 11	> 12	> 13	> 14
Sensitivity	0.75	0.64	0.41	0.23	0.16	0.81	0.68	0.48	0.32	0.26
Specificity	0.32	0.58	0.87	0.90	0.94	0.32	0.50	0.79	0.89	0.95
PPV	0.67	0.74	0.85	0.82	0.82	0.40	0.43	0.56	0.63	0.73
NPV	0.42	0.47	0.45	0.40	0.38	0.75	0.74	0.73	0.70	0.70
LR+	1.11	1.53	3.18	2.58	2.49	1.19	1.35	2.26	3.01	4.82
LR−	0.78	0.62	0.68	0.83	0.90	0.60	0.65	0.66	0.76	0.78
% correctly classified	60.0	62.1	57.5	48.3	43.7	49.4	56.3	68.9	68.9	70.1

*AUC*, Area under curve; *CI*, confidence interval; *PPV*, positive predictive value; *NPV*, negative predictive value; *LR*+, positive likelihood ratio; *LR*-, negative likelihood ratio; * *p* < 0.05

Baseline AHI had significant predictive value by both criteria (Tables 1 and 3). By adding the baseline AHI to the CPAPmax in a simple prediction model, the ROC AUC (95% CI) increased to 0.70 (0.59–0.81, *p* = 0.002) and 0.73 (0.61–0.85, *p* < 0.001) by criterion 1 and 2, respectively (Fig. 1).

A prediction model using a CPAPmax > 12 cm H_2_O and baseline AHI ≥ 30 as cutoff values had a positive predictive value (PPV) of 1.0 for OA non-response both by criterion 1 and criterion 2 (Table 5).

**Table 5:** Positive predictive values for OA treatment failure combining maximum CPAP pressure and baseline AHI by criterion 1 AHI < 5 and criterion 2 5 ≤ AHI < 10 and > 50% AHI reduction (C1/C2)

		AHI at baseline				
		≥ 20	≥ 25	≥ 30	≥ 35	≥ 40
Maximum CPAP pressure (H_2_O)	> 10	0.74/0.50	0.81/0.62	0.82/0.65	0.92/0.77	0.91/0.82
	> 11	0.81/0.54	0.91/0.73	0.92/0.85	1.00/0.91	1.00/1.00
	> 12	0.95/0.67	1.00/0.86	1.00/1.00	1.00/1.00	1.00/1.00
	> 13	0.92/0.75	1.00/0.89	1.00/1.00	1.00/1.00	1.00/1.00
	> 14	1.00/0.88	1.00/1.00	1.00/1.00	1.00/1.00	1.00/1.00

## Discussion

In this study, we sought to investigate the relationship between auto-CPAP pressures and OA treatment outcome in moderate to severe obstructive sleep apnea patients all non-adherent to CPAP treatment. Our findings indicate that the pressures delivered from auto-CPAP machines were associated with the subsequent OA treatment outcome. Combining the easily available maximum pressure data from the CPAP software with the AHI from the baseline respiratory polygraphy, cutoff values with high predictive values for OA treatment failure were identified.

The results are in accordance with the findings in the previously mentioned studies [14–16]. However, as in the Canadian study [16], the correlation between CPAP pressures and OA treatment outcome was not as strong as reported in the Japanese and Australian populations [14, 15]. There might be several reasons for this.

First, we have to consider the possible effect of the differences in the pressure modes used. All patients in the present study used CPAP in auto pressure mode, whereas fixed pressure CPAP was applied for treatment in the Japanese and Australian studies. The fixed pressure level was defined either by manual in-laboratory titration [14] or the 95th percentile pressure from auto-CPAP [15] and confirmed to be therapeutic with polysomnography. In the Canadian study [16], the pressure data were retrieved from a variety of commercially available CPAP machines, mainly of auto-adjusting type, using the 90th percentile pressure. Auto-CPAP devices adjust the treatment pressure based on feedback from various patient measures (airflow, pressure fluctuations, measures of airway resistance), applying the lowest effective pressure to splint the airways open. The mode takes into account the pressure variation during sleep over a given period of time. A single-night titration, determining the fixed pressure requirements either manually or by auto-CPAP, may be affected by an inadequate sampling of sleep position and quality [21]. Studies comparing the different modes in respect to pressure levels have shown that the therapeutic pressures are lower using an automatic mode [22]. The therapeutic pressure requirements also vary with the CPAP manufacturer and device algorithm [21]. Consequently, the differences in the pressures reported in various publications may depend on the choice of CPAP study design.

Second, in the Australian and Japanese studies [14, 15], the inclusion criteria comprised adherence to CPAP, indicating that the participants were able to sleep most of the night during treatment. Thus, breathing cessations demanding higher therapeutic pressures will occur more frequently in the CPAP adherent, sleeping patient compared to the partially awake patient struggling with mask and machine acceptance. In our study, all patients were non-adherent to CPAP therapy. However, this does not necessarily mean that they were truly non-compliant. The reasons for discontinuing CPAP treatment were diverse, some of which might relate to high pressure levels, whereas others could be “pressure independent,” e.g., lack of motivation. To receive reimbursement for OA therapy in Norway, non-adherence to previous CPAP therapy is required, which might contribute to a higher fraction of unmotivated CPAP users, using the machine for only a short period of time mostly being awake. One might speculate how this would affect the CPAP pressures recorded, most likely reducing the pressure variations.

Third, the two studies [14, 15] also included patients with mild sleep apnea in addition to moderate and severe disease, as opposed to our study, which included only patients with moderate or severe sleep apnea. Still, the baseline AHI was higher in both studies, indicating a wider range of AHI, with more patients in each end of the severity scale. An elevated AHI at baseline is associated with an increased risk of OA treatment failure [23]. Baseline AHI has also been included in mathematical equations to predict the CPAP pressure needed to alleviate airway obstruction; the higher the baseline AHI, the higher the therapeutic CPAP pressure [24]. Consequently, the pressure difference observed between responders and non-responders could be more pronounced in the referred studies than in our population.

The study populations also differ in other ways. The Japanese population had a lower BMI and included only males [14], whereas the Australian population was younger [15], compared to the present study. In the Canadian study [16], the patients were in many respects similar to ours. Accordingly, different patient selection might explain the moderate result variation between studies examining the relationship concerning CPAP pressures and OA treatment efficacy.

Sleep apnea is a heterogeneous disorder with several phenotypes related to anatomical and non-anatomical factors [25, 26]. Reliable and simple methods for identification of these phenotypes could allow for future customized therapy [26]. However, given the diverse nature of OSA and based on the findings in this and previous studies [11–13], it seems unlikely that a single clinical or polysomnographic variable is sufficient to predict OA treatment outcome for every patient. A combination of several characteristics might have better predictive ability. Such models should be clinically applicable and aim for a high percentage of correctly classified treatment outcomes. This has been addressed in a previous study, which used models based on patient characteristics and OSA phenotypes [13]. However, no reliable prediction tool was found, and the authors concluded that other prediction methods were needed. A different approach could be to develop models that reliably identify a subset of the OA non-responders, i.e., models with high predictive value for OA non-response. In that way, other effective treatment strategies for these patients would be initiated earlier, saving health care resources. In a prospective study of moderate OSA patients, a combination of Mallampati score of class 4 and BMI > 24 had high predictive value for OA non-response [27]. Using these cutoff levels, about 30% of all non-responders were identified. This coincides with our results. A prediction model combining the maximum CPAP pressures and baseline AHI seemed to identify a phenotypic trait not responding to OA, predicting treatment failure with high accuracy. Other methods that examine the change of upper airways dimensions during mandibular advancement, either by sleep monitoring with the simultaneous use of a remotely controlled mandibular positioner or by endoscopic evaluation, have shown promising results [28, 29], but are invasive and operator dependent and time and cost consuming.

There are other definitions of CPAP adherence than the one used in this study, e.g., ≥ 4 h/night in ≥ 70% of nights [5]. What constitutes optimal use is not known. There seems to be a dose-response relationship between CPAP use and clinical outcome, implying that there is a lower threshold for the duration of therapy, below which the treatment effects disappear [4, 5]. Still, some patients who would otherwise be considered as CPAP non-adherent may profit on usage of shorter duration, without the need for treatment change [18]. Thus, the level of adherence must always be compared to the clinical effect. For the majority to benefit from treatment; however, the CPAP should be used most of the time asleep [30]. A cutoff of at least 4 h use per night has proven valid for various outcome measures [18, 30].

There are limitations and strengths to our study. The data were retrieved retrospectively, making room for bias. However, the information was collected from consecutive patients, using CPAP software and medical records, i.e., reliable sources. The Center for Sleep Medicine in the present study serves as a single regional center, which in addition to diagnosing OSA, also includes initiation of therapy and subsequent follow-up of all patients, even if they initially were evaluated by private health care providers. Thus, the patient assortment should be representative for the diagnosed OSA population. In Norway, CPAP is still the first line of treatment for all severity levels of OSA, and patients get their OA expenses reimbursed only if they fail CPAP therapy. These factors reduce the risk of selection bias.

In conclusion, this study shows that high CPAP maximum pressures are associated with subsequent OA treatment failure in non-compliant auto-CPAP users suffering from moderate to severe OSA. The predictive ability was further improved when pressure was combined with the baseline AHI, reflecting disease severity. A prediction model using a combination of CPAP maximum pressure > 12 cm H_2_O and a baseline AHI ≥ 30 as cutoff values had very high predictive values in identifying OA non-responders, but needs prospective validation.
